# Long‐term mortality after tuberculosis treatment among persons living with HIV in Haiti

**DOI:** 10.1002/jia2.25721

**Published:** 2021-07-08

**Authors:** Yvetot Joseph, Zhiwen Yao, Akanksha Dua, Patrice Severe, Sean E Collins, Heejung Bang, Marc Antoine Jean‐Juste, Oksana Ocheretina, Alexandra Apollon, Margaret L McNairy, Kathryn Dupnik, Etienne Cremieux, Anthony Byrne, Jean W Pape, Serena P Koenig

**Affiliations:** ^1^ Haitian Study Group for Kaposi’s Sarcoma and Opportunistic Infections (GHESKIO) Port‐au‐Prince Haiti; ^2^ Analysis Group Boston MA USA; ^3^ Division of Biostatistics Department of Public Health Sciences University of California Davis CA USA; ^4^ Center for Global Health Department of Medicine Weill Cornell Medical College New York NY USA; ^5^ Department of Medicine University of New South Wales Sydney NSW Australia; ^6^ Division of Global Health Equity Brigham and Women’s Hospital Harvard Medical School Boston MA USA

**Keywords:** HIV, AIDS, tuberculosis, long‐term mortality, opportunistic infections

## Abstract

**Introduction:**

Long‐term mortality among TB survivors appears to be higher than control populations without TB in many settings. However, data are limited among persons with HIV (PWH). We assessed the association between cured TB and long‐term mortality among persons with PWH in Haiti.

**Methods:**

A prospective cohort of PWH from the CIPRA HT‐001 trial was followed from study enrolment (August 2005 to July 2008) to study closure (December 2018) to compare mortality between participants with and without TB. The index date for the survival analysis was defined as 240 days after TB diagnosis or randomization date. Time to death was described using Kaplan–Meier curves, and log‐rank tests were used to compare time to death between the TB and no‐TB cohorts. The association between TB and long‐term mortality was estimated with multivariable Cox models.

**Results:**

Of the 816 participants in the CIPRA HT‐001 trial, 77 were excluded for a history of TB prior to study enrolment and 31 were excluded due to death or attrition prior to the index date, leaving 574 in the no‐TB and 134 in the TB cohort. Twenty‐four (17.9%) participants in the TB and 48 (8.4%) in the no‐TB cohort died during follow‐up. Five and 10‐year mortality rates were 14.2% and 17.9% respectively, in the TB cohort, and 6.1% and 8.4% in the no‐TB cohort. In Kaplan–Meier analysis, participants in the TB cohort had a significantly shorter time to death (log‐rank *p* < 0.001). In multivariable analysis, TB treatment was the only predictor of mortality (HR: 2.78; 95% CI: 1.61, 4.79). Sensitivity analyses, which included only baseline TB cases, an index date of two years after TB diagnosis, and study enrolment and case‐control matching yielded results that were consistent with primary analyses.

**Conclusions:**

PWH who are successfully treated for TB have higher long‐term mortality than those who are never diagnosed with TB, even after accounting for acute TB‐related mortality. A better understanding of the underlying mechanisms associated with TB sequelae is critically needed to guide specific interventions. Until then, more aggressive measures for health promotion and disease prevention are essential to improve long‐term survival for PWH after TB treatment.

## 1 Introduction

Although tuberculosis (TB) is a preventable and curable disease, it remains the leading cause of death among persons with HIV (PWH), accounting for about one‐third of all reported HIV‐related deaths [1, 2]. However, this does not account for TB‐related deaths that occur after treatment completion. A growing body of published reports from largely HIV‐negative populations has found that TB survivors continue to have an elevated long‐term mortality rate, compared with the general population [3, 4, 5, 6, 7, 8, 9, 10]. Data on long‐term mortality after successful TB treatment in PWH are limited, but also suggest that both mortality from active infection and deaths from persistent sequelae of TB disease after cure contribute to TB‐related mortality. A cohort study from Vietnam, which included a subset of PWH, found that two‐thirds of deaths occurred after TB treatment completion [3]. A retrospective analysis of the Caribbean, Central and South America Network for HIV Epidemiology (CCASAnet) database found higher rates of long‐term mortality in PWH after TB treatment completion, compared to those without TB [11]. However, the CCASAnet study was limited by retrospective design, inability to determine TB treatment outcome and lack of data to adjust for clinical factors in a heterogenous population.

The goal of this study was to evaluate the impact of TB on long‐term mortality in a prospectively followed cohort of PWH with similar socio‐economic and clinical status. We compared the mortality rates between PWH with and without TB, using 14 years of longitudinal data from the CIPRA HT‐001 trial cohort.

## 2 Methods

### 2.1 Study setting

This study was conducted at the Haitian Group for the Study of Kaposi’s Sarcoma and Opportunistic infections (GHESKIO) in Port‐au‐Prince, Haiti. GHESKIO is a Haitian nongovernmental organization and the largest provider of HIV care in the Caribbean, treating up to 700 patients per day for HIV and/or TB.

### 2.2 Overview of the CIPRA HT‐001 study

**Figure 1:**
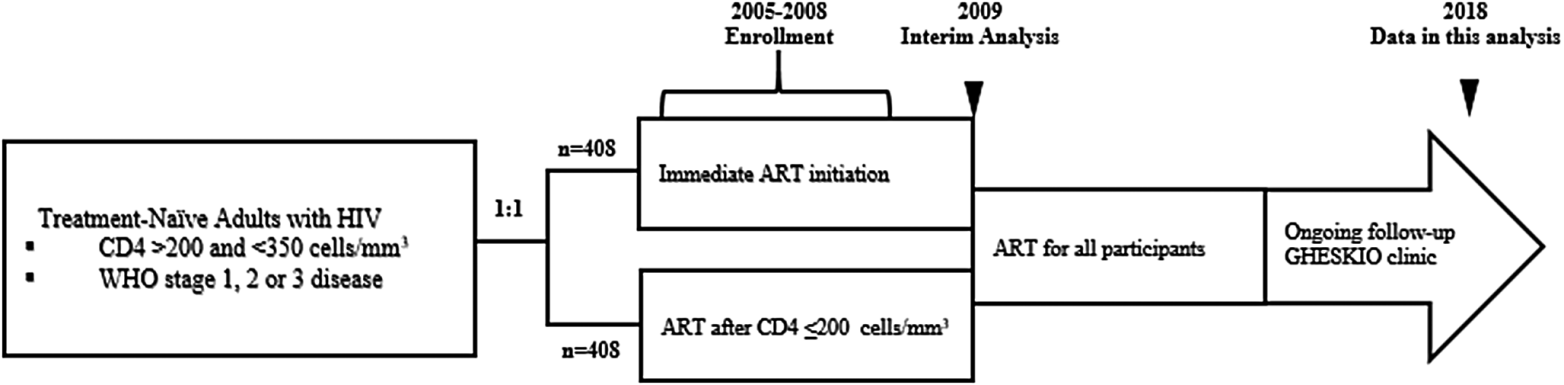
CIPRA HT‐001 original study design and observational follow‐up.

CIPRA HT‐001 was an open‐label randomized trial that was conducted at GHESKIO [12]. The trial enrolled 816 antiretroviral therapy (ART)‐naïve PWH ≥18 years of age with CD4 cell count between 200 and 350 cells/mm^3^, and no history of an AIDS‐defining illness (World Health Organization [WHO] stage 4) between August 2005 and July 2008. Participants were randomly assigned either to initiate ART within two weeks of enrolment (early treatment group), or to start ART when they developed an AIDS‐defining illness or their CD4 cell count reached <200 cells/mm^3^ (deferred treatment group), which was the standard of care at the time (Figure 1) [12].

Demographic and clinical characteristics were comparable between the early and deferred treatment groups at randomization. The median age of participants was 40 years, and 470 (58%) were women. The median CD4 cell count was 281 cells/mm^3^. Detailed results have been published previously [12]. At the second interim analysis, which included data accumulated up to May 1, 2009, the trial crossed the prespecified stopping boundary for a difference in survival between the groups, and the data safety monitoring board recommended that the trial be stopped, and all participants initiate ART. The median follow‐up time from enrolment to study stop date was 21 months. There were 23 deaths in the deferred group, compared with six in the early treatment group (hazard ratio [HR]: 4.0 (95% confidence interval [CI]: 1.6 to 9.8). There was also a higher incidence of TB in the deferred group (n = 36) compared to the early treatment group (n = 18) (HR: 2.0; 95% CI: 1.2 to 3.6).

### 2.3 CIPRA HT‐001 study intervention and subsequent follow‐up

During the CIPRA HT‐001 trial, and throughout the post‐study follow‐up period, participants in both groups received a package of services which were similar to the care provided for all PWH at GHESKIO at the time. At enrolment, participants were screened for latent TB using purified protein derivative (PPD) skin tests; those with a positive PPD, defined as ≥5 mm, were prescribed daily isoniazid. First‐line ART included efavirenz, lamivudine and zidovudine until 2010, when zidovudine was replaced with tenofovir disoproxil fumarate. All participants also received trimethoprim‐sulfamethoxazole prophylaxis.

Participants with symptoms of pulmonary TB received a chest x‐ray and sputum testing for tuberculosis. Initially, sputum testing included modified Ziehl–Neelsen stain and culture for *Mycobacterium tuberculosis* using the BACTEC MGIT 960 system (Becton Dickinson, Franklin Lakes, NJ, USA). In 2010, Xpert MTB/RIF testing (Cepheid, Sunnyvale, CA, USA) became available. Participants who were diagnosed with TB were treated with standard first‐line rifampin‐based therapy, followed by secondary isoniazid prophylaxis. Participants with recurrent TB or drug‐resistant TB were treated according to WHO guidelines, which evolved over time.

### 2.4 Tuberculosis definitions

TB was defined according to the definition of the American Thoracic Society, as described in previous reports [13, 14]. For diagnosis, the required criteria included symptoms of active TB and bacteriologic confirmation of disease (positive sputum smear, Xpert MTB/RIF test, or culture) or chest X‐ray findings that were highly suggestive of active TB and clinical response to anti‐tuberculosis treatment. Participants were considered to have baseline TB if they were receiving TB treatment at enrolment, or diagnosed with TB within the subsequent 60 days. Incident TB was defined as newly diagnosed TB which occurred after this period.

### 2.5 Study population for the current analysis

Participants with a self‐reported or documented history of past TB (prior to randomization) but a negative diagnosis at baseline were excluded from the current analysis because of the inability to confirm their TB diagnosis in accordance with the consistent standards applied to those included in the analysis cohort. The remaining participants were divided into two cohorts, based on TB status: (1) TB cohort (baseline or incident TB) and (2) no‐TB cohort (participants never diagnosed with TB). To account for acute mortality due to TB or other aetiology, participants who died or were lost to follow‐up (LTFU) within 240 days after TB diagnosis or after randomization were excluded from analyses. This 240‐day window period was chosen because standard TB treatment should be completed within this time frame [15].

### 2.6 Statistical analyses

Demographic, clinical and laboratory information were extracted from the study database and GHESKIO electronic medical record (EMR) and exported into SAS Enterprise Guide Version 7.1 (Statistical Analysis System, Cary, NC, USA) and R (R: A Language and Environment for Statistical Computing, Vienna, Austria). Baseline characteristics were summarized for each cohort using medians and interquartile ranges (IQR) for continuous variables and counts and percentages for categorical variables. For participants with baseline TB and those who were never diagnosed with TB, the index date for the survival analysis was defined as 240 days after randomization date. For participants with incident TB, the index date was defined as 240 days after TB diagnosis. Participants were followed from the index date (start of follow‐up) through the date of death or end of follow‐up, whichever came first, and censored upon either LTFU or end of data availability (December 2018).

Time to death was described using Kaplan–Meier curves for both the TB and no‐TB cohorts. Log‐rank tests were used to compare the time to death between the two cohorts. Univariable and multivariable Cox Proportional Hazards models were used to estimate the association between TB and hazard of death. All covariates of interest, including age, sex, marital status, income and education levels, body mass index (BMI), CD4 count at ART initiation and randomization group (early or deferred ART) were included in both the univariable and multivariable models. Hazard ratios and associated confidence intervals from the univariable and multivariable models were calculated and reported.

### 2.7 Sensitivity analyses

Three sets of sensitivity analyses were conducted. First, the main analyses were repeated among participants with baseline TB or no TB, excluding the incident TB participants; the index date was 240 days after randomization. Next, the main analyses (with the inclusion of participants with baseline and incident TB) were repeated with a conservative two‐year window period for estimated TB treatment period (rather than 240 days) to reduce the likelihood of misclassifying mortality related to active or incompletely treated TB. In this analysis, the index date was set to two years after randomization date (for participants with baseline TB or no TB) or two years after TB diagnosis (for participants with incident TB). Finally, a matched analysis was conducted to avoid potential bias due to shorter follow‐up time in the incident TB cohort. The survival analyses were repeated in a matched cohort based on the date of TB diagnosis (for the TB cohort) and the date of randomization (for the no‐TB cohort). A many‐to‐one greedy nearest neighbour match was performed to match each participant in the TB cohort with four in the no‐TB cohort, with a window period of up 365 days between TB diagnosis and date of randomization. Many‐to‐one match was performed to maximize sample size, due to the relatively small number of events.

### 2.8 Ethical considerations

The CIPRA HT‐001 study and subsequent analyses were approved by the institutional review boards at all participating institutions. All participants provided written informed consent.

## 3 Results

**Figure 2:**
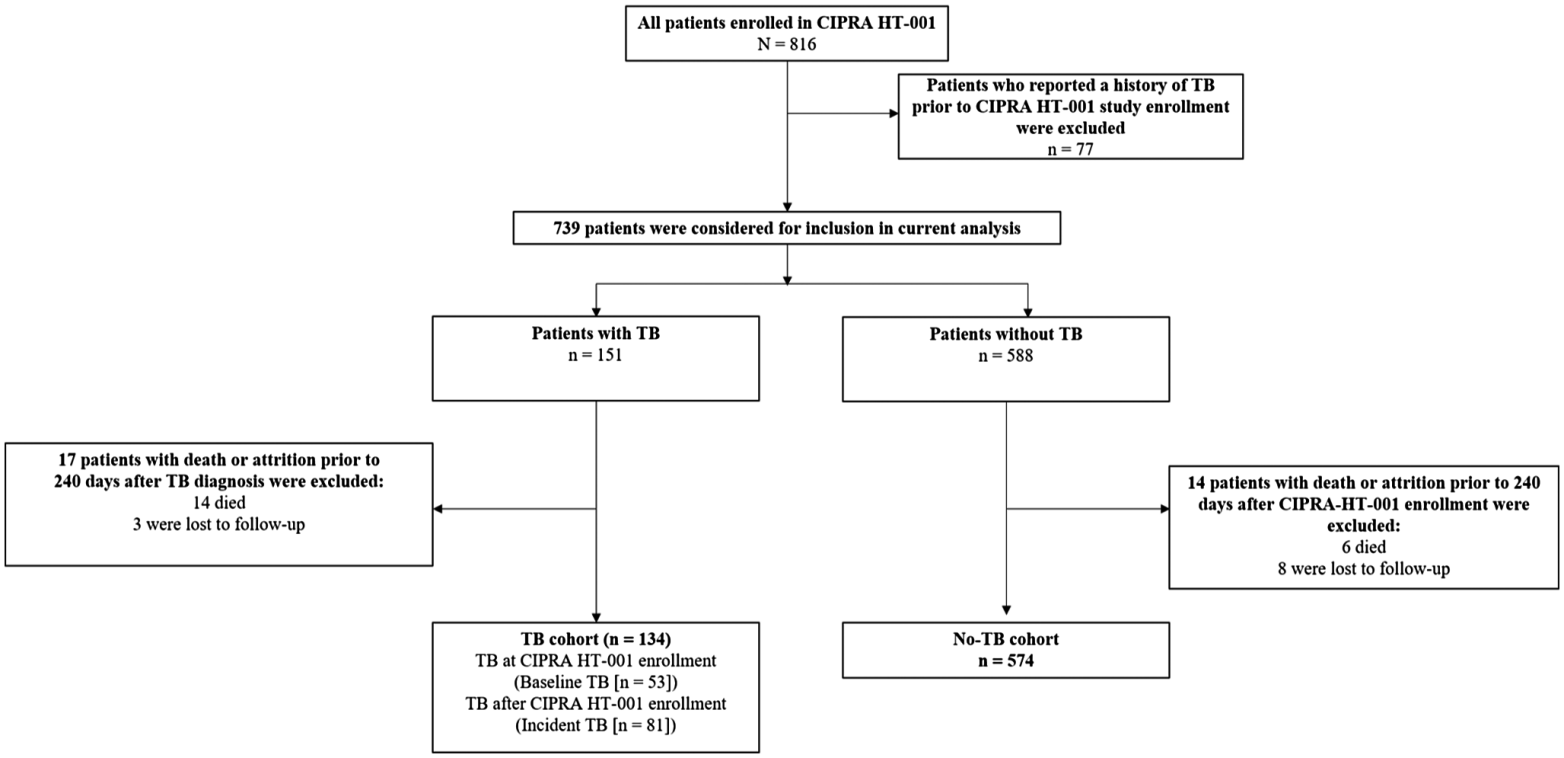
Study population for current analysis.

**Figure 3:**
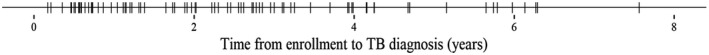
Time from CIPRA HT‐001 enrollment to TB diagnosis for patients with incident TB.

Of the 816 participants in the CIPRA HT‐001 trial, 77 were excluded for reported history of TB prior to study enrolment. Of the 739 remaining participants, 14 in the TB cohort and six in the no‐TB cohort were excluded due to death within 240 days after TB diagnosis or date of randomization. Three participants in the TB cohort and eight in the no‐TB cohort were excluded due to LTFU within the same period (Figure 2). The final analyses set therefore included 708 participants, 574 in the no‐TB cohort and 134 in the TB cohort (baseline TB: 53; incident TB: 81). Among those with incident TB, the median time from study enrolment to TB diagnosis was 2.0 years (IQR: 0.9, 3.5) (Figure 3). Among the 134 participants with TB, only 1 (<1%) was known to have rifampin resistance.

Among the 708 participants included in the analysis, the median age at enrolment was 40 years (IQR: 33, 46) and 410 (58%) were women. Of the 134 participants in the TB cohort, 73 (54%) (baseline TB: 20; incident TB: 53) were originally randomized to the deferred ART group and 61 (46%) (baseline TB: 33; incident TB: 28) to the early ART group. Of the 574 in the no‐TB group, 277 (48%) were randomized to the deferred ART group and 297 (52%) to the early ART group. Demographic characteristics, including age, sex, education, income and marital status, were similar between patients in the TB and no‐TB cohorts (Table 1). CD4 cell counts at enrolment were also similar between the TB and no‐TB cohorts, with median values of 276 cells/mm^3^ (IQR: 248, 309) and 276 cells/mm^3^ (IQR: 241, 312) respectively (*p* = 0.834). Among the participants who initiated ART, CD4 cell counts at ART initiation were lower in the TB cohort compared to the no‐TB cohort, with median values of 247 cells/mm^3^ (IQR: 171, 290) and 257 cells/mm^3^ (IQR: 205, 302) respectively (*p* = 0.026). Additionally, participants in the TB cohort had lower BMI compared to those in the no‐TB cohort at initial randomization (19.9 vs. 21.5; *p* < 0.001), and were more likely to have been categorized as WHO clinical stage 3 at that time (44% vs. 3%; *p* < 0.001), because pulmonary TB is a WHO stage 3 condition. Eight patients were diagnosed with diabetes at baseline or during the study period; 5 (0.9%) were in the no TB cohort and 3 (2.2%) were in the TB cohort (*p* = 0.177).

**Table 1:** Demographic and clinical variables (n = 708)

Characteristic	TB Cohort (n = 134)	No‐TB Cohort (n = 574)	*p*‐value
Age at study entry (years) – median (IQR)	38 (33, 45)	40 (33, 47)	0.182
Female sex – no. (%)	71 (53)	339 (59)	0.234
Education – no. (%)			
No school	41 (31)	170 (30)	0.784
Primary school	37 (28)	176 (31)	
Secondary school or more	56 (42)	228 (40)	
Annual income <$100/year – no. (%)	119 (89)	510 (89)	1.000
Living with spouse or partner – no. (%)	53 (40)	236 (41)	0.815
World Health Organization stage at randomization – no. (%)			
World Health Organization stage 1	24 (18)	226 (40)	<0.001
World Health Organization stage 2	51 (38)	329 (57)	
World Health Organization stage 3	59 (44)	19 (3)	
CD4 count at study entry (cells/mm^3^) – median (IQR)	276 (248, 309)	276 (241, 312)	0.834
CD4 count at ART initiation (cells/mm^3^) – median (IQR)	247 (171, 290)	257 (205, 302)	<0.026
Body mass index at study entry (kg/m^2^) – median (IQR)	19.9 (18.4, 22.2)	21.5 (19.6, 23.9)	<0.001
CIPRA HT‐001 randomization group – no. (%)			
Early ART group	61 (46)	297 (52)	0.230
Deferred ART group	73 (55)	277 (48)	

**Figure 4:**
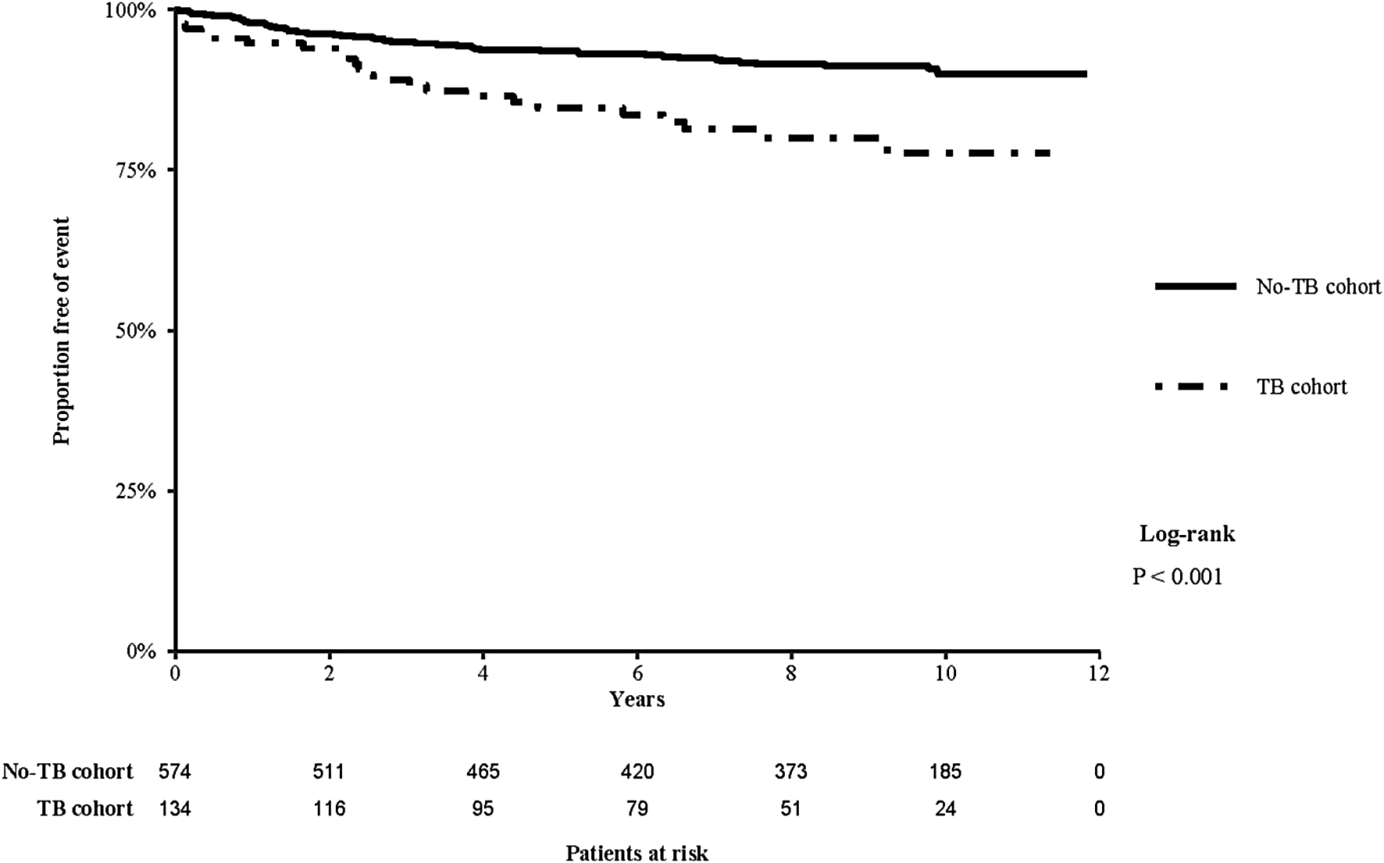
Kaplan–Meier estimates of survival in the TB and No‐TB cohorts (starting from 240 days after TB diagnosis or randomization date).

Over the study period, the median follow‐up time from the index date (240 days after randomization or TB diagnosis) was 9.1 years (IQR: 5.1, 10.1) for the overall cohort, 7.1 years (IQR: 3.5, 9.2) for the TB cohort and 9.2 years (IQR: 5.5, 10.2) for the no‐TB cohort. Twenty‐four (17.9%) of participants in the TB cohort and 48 (8.4%) in the no‐TB cohort died during follow‐up. The median age at death was 42.2 years (IQR: 38.1, 50.9) for the TB cohort and 49.8 years (IQR: 43.2, 55.2) for the no‐TB cohort. Five and 10‐year mortality rates were 14.2% and 17.9%, respectively, in the TB cohort, and 6.1% and 8.4% in the no‐TB cohort. In Kaplan–Meier analysis (Figure 4), participants in the TB cohort had a significantly shorter time to death (log‐rank *p* < 0.001).

Among the 133 participants who were not known to have rifampin‐resistant TB, a total of six were treated for a second episode of TB, during 945 person‐years at risk (PYAR). The incidence rate of recurrent TB was 6.3/1000 PYAR. Four of these patients died during follow‐up.

In the univariable analysis, TB, older age and secondary school were associated with mortality (Table 2). In the multivariable analysis, TB (HR: 2.78; 95% CI: 1.61, 4.79) was the only significant predictor of mortality.

**Table 2:** Predictors of mortality during follow‐up: univariable and multivariable Cox Proportional Hazards regression analyses

Variable (reference category)	Univariable analysis		Multivariable analysis	
	HR (95% CI)	*p*‐value	HR (95% CI)	*p*‐value
TB (vs. no TB)	2.44 (1.49, 3.99)	<0.001	2.78 (1.61, 4.79)	<0.001
Age, per decade	1.37 (1.08, 1.73)	0.008	1.25 (0.96, 1.63)	0.092
Education				
Primary (vs. no school)	0.62 (0.35, 1.10)	0.102	0.78 (0.42, 1.45)	0.436
Secondary school (vs. no school)	0.51 (0.30, 0.89)	0.017	0.57 (0.30, 1.10)	0.093
Living with spouse or partner (vs. single)	1.47 (0.93, 2.34)	0.101	1.15 (0.68, 1.94)	0.599
Body mass index (kg/m^2^)	0.94 (0.88, 1.01)	0.099	0.99 (0.91, 1.07)	0.708
Female sex (vs. male sex)	0.74 (0.47, 1.18)	0.208	0.75 (0.43, 1.32)	0.318
Annual income <$100/year	1.43 (0.62, 3.29)	0.406	1.72 (0.60, 4.93)	0.310
Early randomization group (vs. deferred group)	0.89 (0.56, 1.42)	0.636	1.49 (0.81, 2.75)	0.201
CD4 count at ART initiation, per 50 cells	0.95 (0.80, 1.12)	0.546	0.9 (0.73, 1.11)	0.318

### 3.1 Sensitivity analyses

Sensitivity Analysis 1 included only the subgroup of 53 participants with baseline TB and 574 with no TB (5242 patient‐years of follow‐up). Five and 10‐year mortality rates were 11.3% and 18.9%, respectively, in the baseline TB cohort, and 6.1% and 8.4% in the no‐TB cohort. In the Kaplan–Meier survival analysis (Figure S1), participants in the baseline TB cohort had a shorter time to death (log‐rank *p* = 0.006), consistent with the primary analysis. In the multivariable analysis, TB (HR 2.48; 95%: 1.11, 5.51) and older age (HR [per decade]: 1.47 (95% CI: 1.10, 1.98) were associated with mortality (Table S1).

In Sensitivity Analysis 2, the index date was defined as two years after CIPRA HT‐001 enrolment (for participants with baseline TB or no TB) or two years after TB diagnosis (for participants with incident TB). Consistent with the primary analysis, five and ten‐year mortality rates were 11.7% and 14.2%, respectively, in the TB cohort, and 4.3% and 5.9% in the no‐TB cohort. In the Kaplan–Meier survival analysis (Figure S2), participants in the TB cohort had a shorter time to death (log‐rank *p* < 0.001). In multivariable analysis, TB (HR: 2.89: 95% CI: 1.56, 5.34) and older age (HR [per decade]: 1.35; 95% CI: 1.01, 1.81) were associated with mortality (Table S2).

In Sensitivity Analysis 3, patients in the TB cohort were matched to patients in the no‐TB cohort based on the date of TB diagnosis (TB group) and the date of enrolment in the study (no‐TB group). A total of 450 patients were included in this sensitivity analysis (TB: 90; no‐TB: 360), and contributed to 3695 patient‐years of follow‐up. The median time from study enrolment to TB diagnosis among patients with incident TB was 0.7 years (IQR: 0.6, 1.2). In Kaplan–Meier survival analysis (Figure S3), participants in the TB cohort had a shorter time to death (log‐rank *p* = 0.005), which was consistent with the primary analysis. In multivariable analysis, TB (HR: 2.76: 95% CI: 1.32, 5.78) and older age (HR [per decade]: 1.44; 95% CI: 1.00, 2.06) were associated with mortality (Table S3).

## 4 Discussion

The results of this study show that among PWH, co‐infection with TB disease leads to increased long‐term mortality despite successful TB treatment. In this study, with up to 14 years of follow‐up data from the CIPRA HT‐001 trial cohort, 17.9% of participants with baseline or incident TB died after successful completion of TB treatment, compared with 8.4% of those who never had TB. In a multivariable analysis which controlled for initial study randomization, intrinsic characteristics and health status, a history of TB was associated with a nearly threefold higher rate of long‐term mortality, starting from the time of TB treatment completion.

The association with TB and long‐term mortality may have been even greater if secondary isoniazid prophylaxis had not been provided. GHESKIO is one of the few health centres to routinely prescribe secondary isoniazid prophylaxis for PWH after completion of TB treatment [16]. In this study, 4.5% of patients in the TB cohort were treated for the second episode of TB (6.3/1000 PYAR). In contrast, about 18% of PWH in a South African cohort had recurrent TB over a similar follow‐up period (22.6/1000 PYAR after successful TB treatment) [17]. A second South African study of similar duration found that 10.9% of patients developed recurrent TB, without stratification by HIV status [18]. Secondary TB preventive therapy in PWH may reduce the incidence of recurrent TB and its associated mortality [19].

Data from other studies on long‐term outcomes after TB treatment among PWH are limited. One retrospective analysis of the CCASAnet database also found higher rates of long‐term mortality in PWH after TB treatment completion, compared to those without TB (10‐year mortality rate of 19.3% vs. 10.6%) [11]. Among largely HIV‐negative populations, long‐term mortality rates are also higher in TB survivors, though most of these analyses were based on population‐level data, and unable to control for potential confounders that increased risk for TB disease and non‐TB‐related mortality [3, 4, 5, 6, 7, 8, 9]. A recent systematic review and meta‐analysis found that pooled standard mortality was nearly threefold higher among individuals post‐TB, compared with the control group [10]. In a large, prospective cohort study from Vietnam, the standard mortality ratio was 4.0 among patients with TB, compared to the control population of people living in the same households [3]. In the studies that reported cause of death after TB cure, the most common causes of death included cardiovascular disease, neoplasms, infections (recurrent TB, complications of HIV, pneumonia), chronic obstructive pulmonary disease (COPD), cirrhosis and renal failure [3, 5, 6, 9].

Data on the cause of death after successful TB treatment in PWH are limited, but it is likely that chronic pulmonary sequelae play a major role. Pulmonary TB is associated with largely irreversible changes to bronchial and parenchymal structures, leading to bronchiectasis, fibrosis and emphysematous changes. Multiple studies in largely HIV‐negative populations have found an association between past TB and COPD [20, 21, 22]. Moreover, HIV is an independent risk factor for COPD, and airflow obstruction is associated with mortality [23]. Most data on COPD in PWH come from high‐income settings, but emerging studies from low‐ and middle‐income countries also demonstrate a high burden of COPD in PWH [24, 25].

Furthermore, PWH are at risk of pneumonia and invasive disease from *Streptococcal pneumoniae*, particularly if they also have COPD [26]. Although the risk is highest with advanced HIV, it remains elevated even in those with well‐controlled disease [27, 28]. Pneumococcal vaccination is recommended for PWH in guidelines of most high‐income countries, but WHO guidelines recommend against it, due to insufficient evidence of benefit [29, 30, 31, 32, 33, 34]. Further data on the contribution of pneumococcal disease to morbidity and mortality in TB survivors with HIV, and potential vaccine efficacy in this highly vulnerable population, are needed.

Behavioural factors, socioeconomic conditions and comorbidities that increase the risk of TB acquisition and premature mortality also likely contribute to poor outcomes after TB treatment [35]. Host‐level factors may also play a role. Persons who have survived one episode of TB are at risk of recurrent TB. In addition, the acquisition of TB is a marker of immune dysfunction, which may also increase the risk of death from other causes [36]. TB co‐infection may contribute to increased HIV viral load and accelerate HIV disease progression.

TB may also cause persistent inflammation and chronic immune activation, further increasing the risk of cardiovascular disease, which is already a major cause of death in PWH [37, 38]. An analysis of PWH enrolled in the Antiretroviral Therapy Cohort Collaboration (ART‐CC) demonstrated that PWH and a history of TB had a higher risk of mortality from cardiovascular, metabolic and non‐AIDS‐defining events, compared to those without TB [36]. In addition, a retrospective cohort study from Taiwan found a 40% increased risk of cardiovascular events in patients with a history of TB [39]. Patients with HIV and TB may receive particular benefits from screening and prevention for cardiovascular disease, including smoking cessation counselling and management of hypertension and hyperlipidaemia.

In spite of the higher mortality in TB survivors, the WHO and other expert committees do not make specific recommendations regarding the management of patients after TB treatment completion. This represents a potential missed opportunity to improve survival in this vulnerable population [1]. Based on the results of this study, we have implemented a more intensive monitoring strategy for PWH who have been treated for TB at GHESKIO, focusing on aggressive health promotion and disease prevention strategies.

Our study is limited by a lack of data on the cause of death; future studies on mechanisms that lead to increased mortality will be critical in guiding further interventions. This study was conducted in a large urban clinic, and all participants had CD4 counts from 200 to 350 cells/mm^3^ at cohort entry, which may limit the generalizability of our findings. However, though the original trial was designed to assess the impact of early versus deferred ART initiation, the study was stopped soon into follow‐up and all participants were treated the same thereafter, which allowed us to draw from a population with very similar health characteristics and control for many variables that are predictive of premature mortality. Moreover, this was a well‐defined population, followed closely for up to 14 years, with a low rate of loss to follow‐up.

## 5 Conclusions

In conclusion, PWH who are successfully treated for TB have higher long‐term mortality than those who are never diagnosed with TB, even after accounting for acute TB‐related mortality. A better understanding of the underlying mechanisms associated with TB sequelae is critically needed to guide specific interventions. Until then, more aggressive measures for health promotion and disease prevention are essential to improve long‐term survival for PWH after TB treatment.

## Competing interests

The authors have declared no conflict of interest.

## Authors’ contributions

YJ, YZ, AD, SC, HB, MM, KD, JWP and SPK involved in the conception of the study. YJ, PS, SC, MJ, OO and AA carried out acquisition of data. YJ, YZ, AD, SC, HB, EC, SPK and JWP involved in data analysis. YJ and SPK carried out first draft of the manuscript. All authors involved in data interpretation, manuscript revision and final approval of the manuscript.

## Supporting information


**Figure S1**. Kaplan–Meier estimates of survival in the baseline TB and No‐TB cohorts (sensitivity analysis 1)
**Figure S2**. Kaplan–Meier estimates of survival in the TB and No‐TB cohorts (sensitivity analysis 2)
**Figure S3**. Kaplan–Meier estimates of survival in the TB and No‐TB cohorts (sensitivity analysis 3)
**Table S1**. Predictors of mortality during follow‐up: univariable and multivariable Cox Proportional Hazards regression analyses (sensitivity analysis 1)
**Table S2**. Predictors of mortality during follow‐up: univariable and multivariable Cox Proportional Hazards regression analyses (sensitivity analysis 2)
**Table S3**. Predictors of mortality during follow‐up: univariable and multivariable Cox Proportional Hazards regression analyses (sensitivity analysis 3)

## References

[1] Global Tuberculosis Report . Geneva: World Health Organization; 2020.; 2020. [cited 2020 Dec 30]. Available from: https://www.who.int/tb/publications/global_report/TB20_Exec_Sum_20201014.pdf.

[2] Global HIV and AIDS statistics — 2020 fact sheet. UNAIDS. [cited 2020 Sep 25]. Available from: https://www.unaids.org/en/resources/fact‐sheet

[3] Fox GJ , Nguyen VN , Dinh NS , Nghiem LPH , Le TNA , Nguyen TA , et al. Post‐treatment mortality among patients with tuberculosis: a prospective cohort study of 10 964 patients in Vietnam. Clin Infect Dis. 2019;68(8):1359–66. 30202910 10.1093/cid/ciy665

[4] Hoger S , Lykens K , Beavers SF , Katz D , Miller TL . Longevity loss among cured tuberculosis patients and the potential value of prevention. Int J Tuberc Lung Dis. 2014;18(11):1347–52. 25299869 10.5588/ijtld.14.0242 PMC5140005

[5] Shuldiner J , Leventhal A , Chemtob D , Mor Z . Mortality after anti‐tuberculosis treatment completion: results of long‐term follow‐up. Int J Tuberc Lung Dis. 2016;20(1):43–8. 26688527 10.5588/ijtld.14.0427

[6] Christensen AS , Roed C , Andersen PH , Andersen AB , Obel N . Long‐term mortality in patients with pulmonary and extrapulmonary tuberculosis: a Danish nationwide cohort study. Clin Epidemiol. 2014;6:405–21. 25419160 10.2147/CLEP.S65331 PMC4235508

[7] Miller TL , Wilson FA , Pang JW , Beavers S , Hoger S , Sharnprapai S , et al. Mortality hazard and survival after tuberculosis treatment. Am J Public Health. 2015;105(5):930–7. 25790407 10.2105/AJPH.2014.302431 PMC4386531

[8] Tocque K , Convrey RP , Bellis MA , Beeching NJ , Davies PD . Elevated mortality following diagnosis with a treatable disease: tuberculosis. Int J Tuberc Lung Dis. 2005;9(7):797–802. 16013777

[9] Blondal K , Rahu K , Altraja A , Viiklepp P , Rahu M . Overall and cause‐specific mortality among patients with tuberculosis and multidrug‐resistant tuberculosis. Int J Tuberc Lung Dis. 2013;17(7):961–8. 23743316 10.5588/ijtld.12.0946

[10] Romanowski K , Baumann B , Basham CA , Ahmad Khan F , Fox GJ , Johnston JC . Long‐term all‐cause mortality in people treated for tuberculosis: a systematic review and meta‐analysis. Lancet Infect Dis. 2019;19(10):1129–37. 31324519 10.1016/S1473-3099(19)30309-3

[11] Koenig SP , Kim A , Shepherd BE , Cesar C , Veloso V , Cortes CP , et al. Increased mortality after tuberculosis treatment completion in persons living with human immunodeficiency virus in Latin America. Clin Infect Dis. 2020;71(1):215–7. 31629369 10.1093/cid/ciz1032 PMC7312222

[12] Severe P , Juste MA , Ambroise A , Eliacin L , Marchand C , Apollon S , et al. Early versus standard antiretroviral therapy for HIV‐infected adults in Haiti. N Engl J Med. 2010;363(3):257–65. 20647201 10.1056/NEJMoa0910370 PMC3676927

[13] Collins SE , Jean Juste MA , Koenig SP , Secours R , Ocheretina O , Bernard D , et al. CD4 deficit and tuberculosis risk persist with delayed antiretroviral therapy: 5‐year data from CIPRA HT‐001. Int J Tuberc Lung Dis. 2015;19(1):50–7. 10.5588/ijtld.14.0217 25519790

[14] Blumberg HM , Burman WJ , Chaisson RE , Daley CL , Etkind SC , Friedman LN , et al. American thoracic society/centers for disease control and prevention/infectious diseases society of America: treatment of tuberculosis. Am J Respir Crit Care Med. 2003;167(4):603–62. 12588714 10.1164/rccm.167.4.603

[15] Ministère de la Santè Publique et de la population (MSPP) . Programme National de Lutte contre la Tuberculose (PNLT): Manuel de Normes de la Tuberculose en Haiti. Port‐au‐Prince, Haiti: MSPP; 2013.

[16] Fitzgerald DW , Desvarieux M , Severe P , Joseph P , Johnson WD Jr , Pape JW . Effect of post‐treatment isoniazid on prevention of recurrent tuberculosis in HIV‐1‐infected individuals: a randomised trial. Lancet. 2000;356(9240):1470–4. 11081529 10.1016/S0140-6736(00)02870-1

[17] Hermans SM , Zinyakatira N , Caldwell J , Cobelens FGJ , Boulle A , Wood R . High rates of recurrent TB disease: a population‐level cohort study. Clin Infect Dis. 2020. 10.1093/cid/ciaa470 PMC8315130 32333760

[18] Marx FM , Dunbar R , Enarson DA , Williams BG , Warren RM , van der Spuy GD , et al. The temporal dynamics of relapse and reinfection tuberculosis after successful treatment: a retrospective cohort study. Clin Infect Dis. 2014;58(12):1676–83. 24647020 10.1093/cid/ciu186

[19] Basu S , Maru D , Poolman E , Galvani A . Primary and secondary tuberculosis preventive treatment in HIV clinics: simulating alternative strategies. Int J Tuberc Lung Dis. 2009;13(5):652–8. 19383201

[20] Pasipanodya JG , Miller TL , Vecino M , Munguia G , Garmon R , Bae S , et al. Pulmonary impairment after tuberculosis. Chest. 2007;131(6):1817–24. 17400690 10.1378/chest.06-2949

[21] Menezes AM , Hallal PC , Perez‐Padilla R , Jardim JR , Muino A , Lopez MV , et al. Tuberculosis and airflow obstruction: evidence from the PLATINO study in Latin America. Eur Respir J. 2007;30(6):1180–5. 17804445 10.1183/09031936.00083507

[22] Byrne AL , Marais BJ , Mitnick CD , Lecca L , Marks GB . Tuberculosis and chronic respiratory disease: a systematic review. Int J Infect Dis. 2015;32:138–46. 25809770 10.1016/j.ijid.2014.12.016

[23] Crothers K , Butt AA , Gibert CL , Rodriguez‐Barradas MC , Crystal S , Justice AC , et al. Increased COPD among HIV‐positive compared to HIV‐negative veterans. Chest. 2006;130(5):1326–33. 17099007 10.1378/chest.130.5.1326

[24] Akanbi MO , Taiwo BO , Achenbach CJ , Ozoh OB , Obaseki DO , Sule H , et al. HIV associated chronic obstructive pulmonary disease in Nigeria. J AIDS Clin Res. 2015;6(5):1000453. 10.4172/2155-6113.1000453 PMC4521629 26236557

[25] Pefura‐Yone EW , Fodjeu G , Kengne AP , Roche N , Kuaban C . Prevalence and determinants of chronic obstructive pulmonary disease in HIV infected patients in an African country with low level of tobacco smoking. Respir Med. 2015;109(2):247–54. 25538018 10.1016/j.rmed.2014.12.003

[26] Garcia Garrido HM , Mak AMR , Wit F , Wong GWM , Knol MJ , Vollaard A , et al. Incidence and risk factors for invasive pneumococcal disease and community‐acquired pneumonia in human immunodeficiency virus‐infected individuals in a high‐income setting. Clin Infect Dis. 2020;71(1):41–50. 31634398 10.1093/cid/ciz728 PMC7312213

[27] Kirwan PD , Amin‐Chowdhury Z , Croxford SE , Sheppard C , Fry N , Delpech VC , et al. Invasive pneumococcal disease in people with human immunodeficiency virus in England, 1999–2017. Clin Infect Dis. 2020. 10.1093/cid/ciaa522 32789498

[28] Collini PJ , Bewley MA , Mohasin M , Marriott HM , Miller RF , Geretti AM , et al. HIV gp120 in the lungs of antiretroviral therapy‐treated individuals impairs alveolar macrophage responses to pneumococci. Am J Respir Crit Care Med. 2018;197(12):1604–15. 29365279 10.1164/rccm.201708-1755OC PMC6006400

[29] Summary of WHO Position Papers ‐ Recommendations for Routine Immunization. Updated April 2019. [cited 2020 Aug11]. Available from: https://www.who.int/immunization/policy/Immunization_routine_table1.pdf?ua=1

[30] Guidelines for Managing Advanced HIV Disease and Rapid Initiation of Antiretroviral Therapy. World Health Organization, 2017. [cited 2019 Sep 1, 2019]. Available from: http://www.who.int/hiv/pub/guidelines/advanced‐HIV‐disease/en/ 29341560

[31] French N , Nakiyingi J , Carpenter LM , Lugada E , Watera C , Moi K , et al. 23‐valent pneumococcal polysaccharide vaccine in HIV‐1‐infected Ugandan adults: double‐blind, randomised and placebo controlled trial. Lancet. 2000;355(9221):2106–11. 10902624 10.1016/s0140-6736(00)02377-1

[32] French N , Gordon SB , Mwalukomo T , White SA , Mwafulirwa G , Longwe H , et al. A trial of a 7‐valent pneumococcal conjugate vaccine in HIV‐infected adults. N Engl J Med. 2010;362(9):812–22. 20200385 10.1056/NEJMoa0903029 PMC2873559

[33] Watera C , Nakiyingi J , Miiro G , Muwonge R , Whitworth JA , Gilks CF , et al. 23‐Valent pneumococcal polysaccharide vaccine in HIV‐infected Ugandan adults: 6‐year follow‐up of a clinical trial cohort. AIDS. 2004;18(8):1210–3. 15166540 10.1097/00002030-200405210-00018

[34] US Department of Health and Human Services . Guidelines for the Prevention and Treatment of Opportunistic Infections in Adults and Adolescents with HIV. Figure: Recommended Immunization Schedule for Adults and Adolescents with HIV Infection. [cited 2020 Aug 1]. Available from: https://aidsinfo.nih.gov/guidelines/html/4/adult‐and‐adolescent‐opportunistic‐infection/365/figure‐‐immunization

[35] Lonnroth K , Jaramillo E , Williams BG , Dye C , Raviglione M . Drivers of tuberculosis epidemics: the role of risk factors and social determinants. Soc Sci Med. 2009;68(12):2240–6. 19394122 10.1016/j.socscimed.2009.03.041

[36] Pettit AC , Giganti MJ , Ingle SM , May MT , Shepherd BE , Gill MJ , et al. Increased non‐AIDS mortality among persons with AIDS‐defining events after antiretroviral therapy initiation. J Int AIDS Soc. 2018;21:25031. 10.1002/jia2.25031 PMC5810321 29334197

[37] Nou E , Lo J , Grinspoon SK . Inflammation, immune activation, and cardiovascular disease in HIV. AIDS. 2016;30(10):1495–509. 27058351 10.1097/QAD.0000000000001109 PMC4889507

[38] Huaman MA , Kryscio RJ , Fichtenbaum CJ , Henson D , Salt E , Sterling TR , et al. Tuberculosis and risk of acute myocardial infarction: a propensity score‐matched analysis. Epidemiol Infect. 2017;145(7):1363–7. 28202093 10.1017/S0950268817000279 PMC5616129

[39] Chung WS , Lin CL , Hung CT , Chu YH , Sung FC , Kao CH , et al. Tuberculosis increases the subsequent risk of acute coronary syndrome: a nationwide population‐based cohort study. Int J Tuberc Lung Dis. 2014;18(1):79–83. 24365557 10.5588/ijtld.13.0288

