# Supplementary material for: Long‐term mortality after tuberculosis treatment among persons living with HIV in Haiti
Source: J Int AIDS Soc. 2021 Jul 8;24(7):e25721. doi: 10.1002/jia2.25721 (PMC8264404; doi:10.1002/jia2.25721)
Supplement: Supplementary file 1 — Figure S1. Kaplan–Meier estimates of survival in the baseline TB and No‐TB cohorts (sensitivity analysis 1) Figure S2. Kaplan–Meier estimates of survival in the TB and No‐TB cohorts (sensitivity analysis 2) Figure S3. Kaplan–Meier estimates of survival in the TB and No‐TB cohorts (sensitivity analysis 3) Table S1. Predictors of mortality during follow‐up: univariable and multivariable Cox Proportional Hazards regression analyses (sensitivity analysis 1) Table S2. Predictors of mortality during follow‐up: univariable and multivariable Cox Proportional Hazards regression analyses (sensitivity analysis 2) Table S3. Predictors of mortality during follow‐up: univariable and multivariable Cox Proportional Hazards regression analyses (sensitivity analysis 3) [file JIA2-24-e25721-s001.docx]

**Supplemental Figure 1.** **Kaplan–Meier Estimates of Survival in the Baseline TB and No-TB Cohorts (Sensitivity Analysis 1)**

**
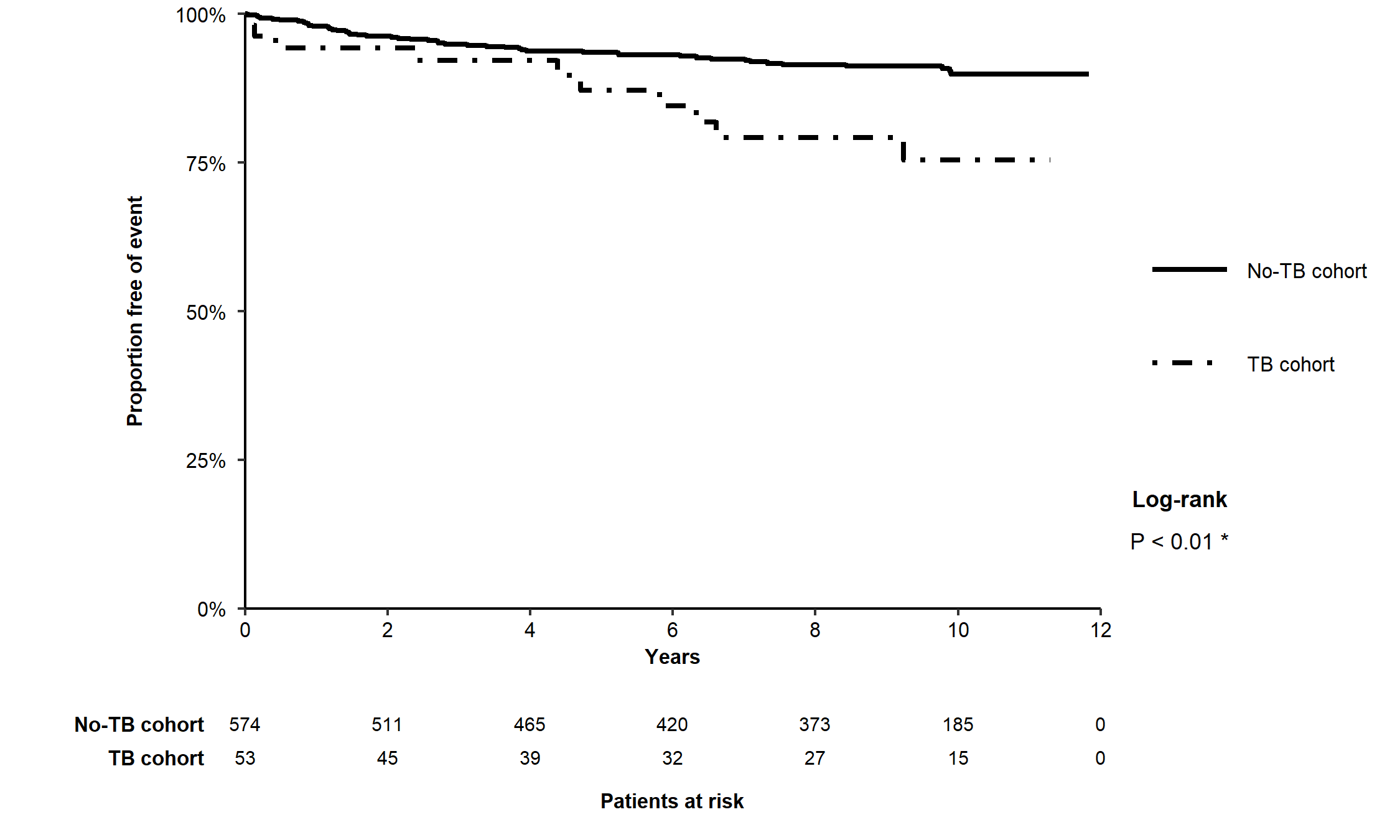
**

**Supplemental Table 1. Predictors of Mortality During Follow-up: Univariable and Multivariable Cox Proportional Hazards Regression Analyses (Sensitivity Analysis 1)**

| **Variable (reference category)** | **Univariable Analysis** | | **Multivariable Analysis** | |
| --- | --- | --- | --- | --- |
|  | HR (95% CI) | p-value | HR (95% CI) | p-value |
| Tuberculosis (vs. no tuberculosis) | 2.51 (1.27, 4.96) | <0.01 | 2.48 (1.11, 5.51) | 0.008 |
| Age, per decade | 1.61 (1.25, 2.07) | <0.001 | 1.47 (1.10, 1.98) | 0.010 |
| Female sex (vs. male sex) | 0.69 (0.41, 1.16) | 0.162 | 0.73 (0.38, 1.41) | 0.352 |
| Education | | | | |
| Primary (vs. no school) | 0.56 (0.30, 1.04) | 0.066 | 0.68 (0.34, 1.37) | 0.284 |
| Secondary school (vs. no school) | 0.43 (0.23, 0.80) | 0.008 | 0.48 (0.22, 1.04) | 0.061 |
| Living with spouse or partner (vs. single) | 1.73 (1.03, 2.90) | 0.037 | 1.37 (0.75, 2.50) | 0.303 |
| Body mass index (kg/m2) | 0.97 (0.90, 1.04) | 0.382 | 1.02 (0.94, 1.11) | 0.674 |
| Annual income <$100/year | 1.37 (0.55, 3.44) | 0.496 | 1.64 (0.48, 5.59) | 0.426 |
| Early randomization group (vs. deferred group) | 0.93 (0.55, 1.55) | 0.771 | 1.69 (0.82, 3.50) | 0.155 |
| CD4 count at ART initiation, per 50 cells | 0.98 (0.80, 1.20) | 0.846 | 0.85 (0.65, 1.10) | 0.220 |

**Supplemental Figure 2.** **Kaplan–Meier Estimates of Survival in the TB and No-TB Cohorts (Sensitivity Analysis 2)**

**
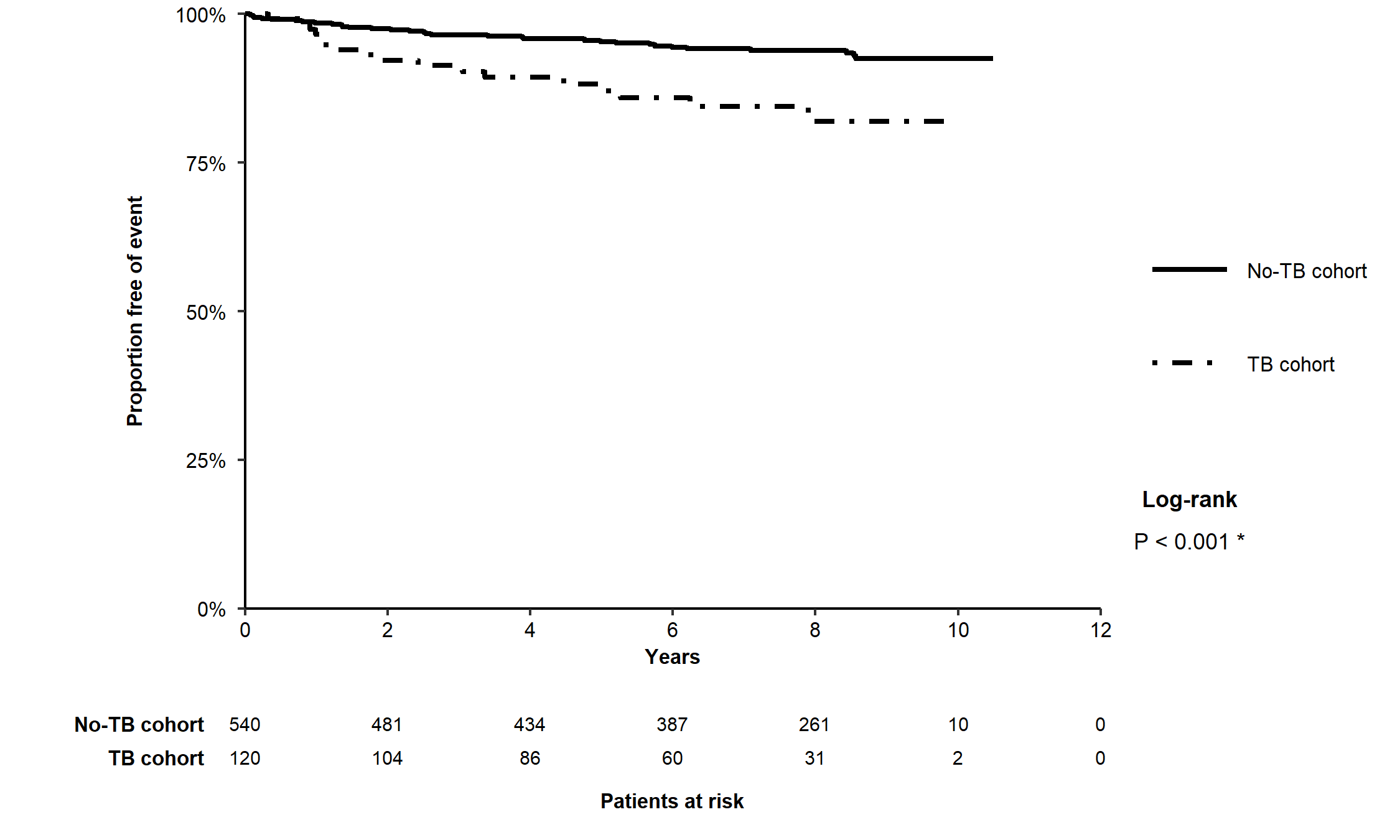
**

**Supplemental Table 2. Predictors of Mortality During Follow-up: Univariable and Multivariable Cox Proportional Hazards Regression Analyses (Sensitivity Analysis 2)**

| **Variable (reference category)** | **Univariable Analysis** | | **Multivariable Analysis** | |
| --- | --- | --- | --- | --- |
|  | HR (95% CI) | p-value | HR (95% CI) | p-value |
| Tuberculosis (vs. no tuberculosis) | 2.71 (1.50, 4.90) | <0.001 | 2.89 (1.56, 5.34) | <0.001 |
| Age, per decade | 1.44 (1.09, 1.90) | 0.0112 | 1.35 (1.01, 1.81) | 0.046 |
| Female sex (vs. male sex) | 0.79 (0.45, 1.38) | 0.408 | 0.70 (0.37, 1.30) | 0.258 |
| Education | | | | |
| Primary (vs. no school) | 0.74 (0.38, 1.42) | 0.363 | 0.95 (0.48, 1.87) | 0.883 |
| Secondary school (vs. no school) | 0.45 (0.22, 0.90) | 0.0236 | 0.57 (0.27, 1.22) | 0.146 |
| Living with spouse or partner (vs. single) | 1.02 (0.58, 1.80) | 0.953 | 0.94 (0.52, 1.72) | 0.843 |
| Body mass index (kg/m^2^) | 0.94 (0.86, 1.02) | 0.142 | 0.98 (0.90, 1.08) | 0.714 |
| Annual income <$100/year | 3.11 (0.76, 12.82) | 0.116 | 2.85 (0.67, 12.12) | 0.157 |
| Early randomization group (vs. deferred group) | 1.25 (0.71, 2.20) | 0.439 | 1.57 (0.79, 3.15) | 0.200 |
| CD4 count at ART initiation, per 50 cells | 0.97 (0.80, 1.17) | 0.722 | 0.90 (0.71, 1.15) | 0.411 |

**Supplemental Figure 3.** **Kaplan–Meier Estimates of Survival in the TB and No-TB Cohorts (Sensitivity Analysis 3)**

**
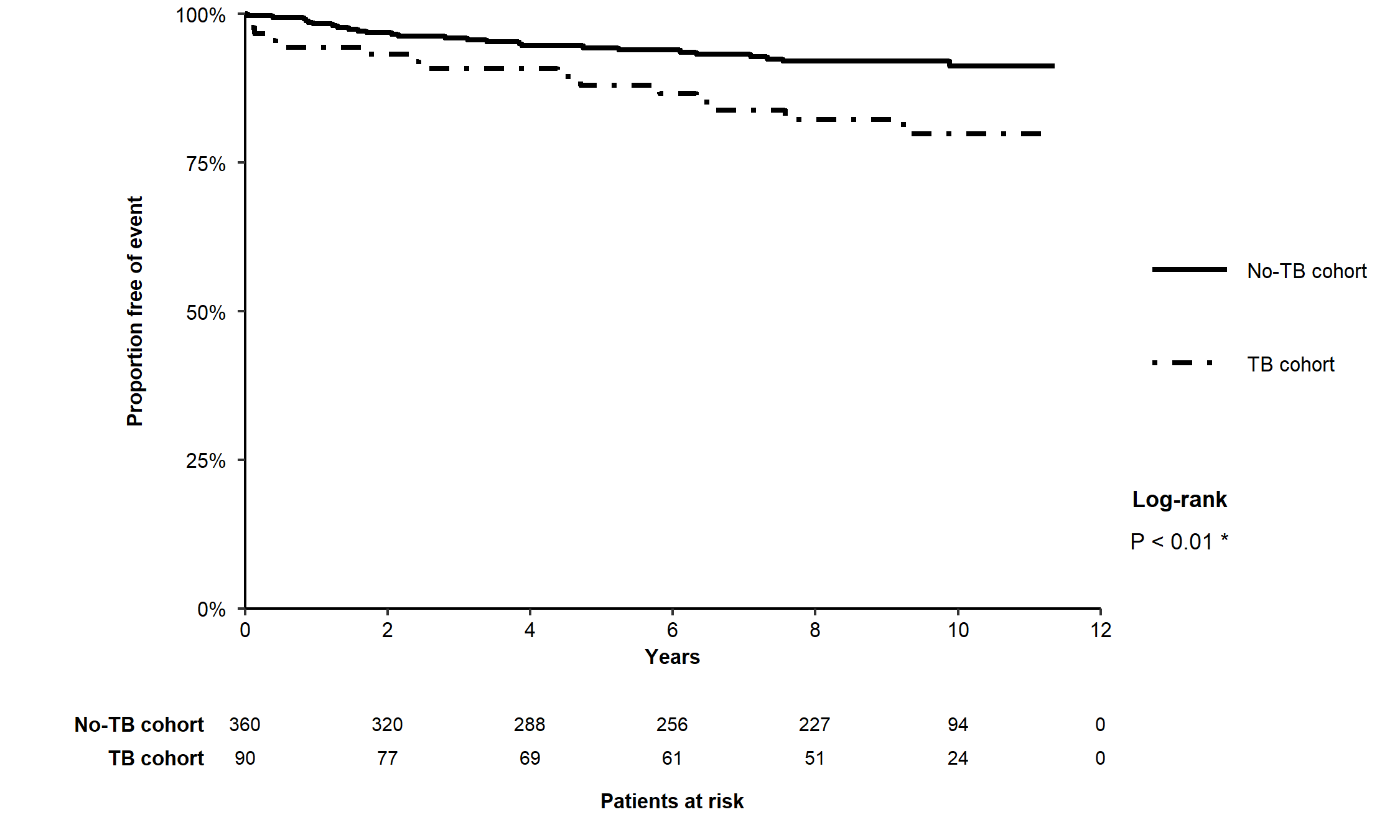
**

**Supplemental Table 3. Predictors of Mortality During Follow-up: Univariable and Multivariable Cox Proportional Hazards Regression Analyses (Sensitivity Analysis 3)**

| **Variable (reference category)** | **Univariable Analysis** | | **Multivariable Analysis** | |
| --- | --- | --- | --- | --- |
|  | HR (95% CI) | p-value | HR (95% CI) | p-value |
| Tuberculosis (vs. no tuberculosis) | 2.43 (1.29, 4.58) | 0.006 | 2.76 (1.32, 5.78) | 0.007 |
| Age, per decade | 1.63 (1.20, 2.21) | 0.002 | 1.44 (1.00, 2.06) | 0.051 |
| Female sex (vs. male sex) | 0.90 (0.49, 1.67) | 0.740 | 0.85 (0.39, 1.81) | 0.667 |
| Education | | | | |
| Primary (vs. no school) | 0.66 (0.33, 1.32) | 0.245 | 0.90 (0.41, 1.96) | 0.785 |
| Secondary school (vs. no school) | 0.30 (0.13, 0.68) | 0.004 | 0.42 (0.16, 1.11) | 0.079 |
| Living with spouse or partner (vs. single) | 1.48 (0.80, 2.74) | 0.207 | 1.20 (0.59, 2.42) | 0.618 |
| Body mass index (kg/m^2^) | 0.98 (0.89, 1.07) | 0.637 | 1.03 (0.93, 1.14) | 0.576 |
| Annual income <$100/year | 1.45 (0.52, 4.07) | 0.482 | 1.39 (0.39, 4.89) | 0.610 |
| Early randomization group (vs. deferred group) | 0.93 (0.50, 1.72) | 0.818 | 1.73 (0.75, 3.97) | 0.196 |
| CD4 count at ART initiation, per 50 cells | 0.92 (0.74, 1.16) | 0.481 | 0.87 (0.65, 1.16) | 0.345 |
